# Genomic Epidemiology of SARS-CoV-2 in Madrid, Spain, during the First Wave of the Pandemic: Fast Spread and Early Dominance by D614G Variants

**DOI:** 10.3390/microorganisms9020454

**Published:** 2021-02-22

**Authors:** Esther Viedma, Elias Dahdouh, José María González-Alba, Sara González-Bodi, Laura Martínez-García, Fernando Lázaro-Perona, Raúl Recio, María Rodríguez-Tejedor, María Dolores Folgueira, Rafael Cantón, Rafael Delgado, Julio García-Rodríguez, Juan Carlos Galán, Jesús Mingorance, on behalf of the SARS-CoV-2 Working Groups

**Affiliations:** 1 Servicio de Microbiología, Hospital Universitario 12 de Octubre and Instituto de Investigación Hospital 12 de Octubre (imas12), 28009 Madrid, Spain; ester.viedma@salud.madrid.org (E.V.); gonbodsar@gmail.com (S.G.-B.); raul.recio@salud.madrid.org (R.R.); lola.folgueira@gmail.com (M.D.F.); rafael.delgado@salud.madrid.org (R.D.); 2 Servicio de Microbiología, Hospital Universitario La Paz and Instituto de Investigación Hospital Universitario La Paz (IdiPAZ), 28046 Madrid, Spain; elie.dahdouh@idipaz.es (E.D.); fernandolazaroperona@gmail.com (F.L.-P.); marirotej@gmail.com (M.R.-T.); jgarcia.hulp@salud.madrid.org (J.G.-R.); 3 Servicio de Microbiología, Hospital Universitario Ramón y Cajal and Instituto Ramón y Cajal de Investigación Sanitaria (IRYCIS), 28034 Madrid, Spain; jmgonzalba@yahoo.es (J.M.G.-A.); laura.martinez.garcia@salud.madrid.org (L.M.-G.); rafael.canton@salud.madrid.org (R.C.); 4 Centro de Investigación Biomédica en Red (CIBER) in Epidemiology and Publich Health, 28029 Madrid, Spain; 5 Red Española de Investigación en Patología Infecciosa (REIPI), 28009 Madrid, Spain

**Keywords:** COVID-19, SARS-CoV-2, genome sequences

## Abstract

Severe acute respiratory syndrome coronavirus 2 (SARS-CoV-2) was first detected in Madrid, Spain, on 25 February 2020. It increased in frequency very fast and by the end of May more than 70,000 cases had been confirmed by reverse transcription-polymerase chain reaction (RT-PCR). To study the lineages and the diversity of the viral population during this first epidemic wave in Madrid we sequenced 224 SARS-CoV-2 viral genomes collected from three hospitals from February to May 2020. All the known major lineages were found in this set of samples, though B.1 and B.1.5 were the most frequent ones, accounting for more than 60% of the sequences. In parallel with the B lineages and sublineages, the D614G mutation in the Spike protein sequence was detected soon after the detection of the first coronavirus disease 19 (COVID-19) case in Madrid and in two weeks became dominant, being found in 80% of the samples and remaining at this level during all the study periods. The lineage composition of the viral population found in Madrid was more similar to the European population than to the publicly available Spanish data, underlining the role of Madrid as a national and international transport hub. In agreement with this, phylodynamic analysis suggested multiple independent entries before the national lockdown and air transportation restrictions.

## 1. Introduction

The pandemic of coronavirus disease 19 (COVID-19) has affected practically all countries around the world. During the first pandemic wave among European countries, Spain reached the highest number of cases and deaths per 100,000 population. The first officially reported COVID-19 case in Spain was detected on January 31st in the Canary Islands in a German citizen related to the Bavarian cluster [1], although the first cases associated to community transmission were detected in Sevilla and Madrid almost one month later (25 February 2020). From there on, the number of daily reported cases increased fast, reaching a peak of 3106 new cases on March 20th and decreasing during April and May to a minimum in June. During this first pandemic wave, Madrid was the Spanish city with the highest number of cases, constituting the epicenter of Spanish COVID-19 pandemic. In fact, in March 11th nearly 50% of all Spanish cases were detected in Madrid. By May 31st 70,264 cases had been confirmed by reverse transcription-polymerase chain reaction (RT-PCR) and more than 8000 deaths had been officially declared. During this period, most hospitals in Madrid became COVID-19 hospitals and had to adapt their procedures to the pandemic situation [2].

As part of the efforts to improve molecular diagnosis and to understand the population dynamics of Severe acute respiratory syndrome coronavirus 2 (SARS-CoV-2) in Madrid during the first pandemic wave, a collaboration was started between our three hospitals, for sequencing the genomes of a representative sample of the virus population circulating in the Madrid region. Several studies have explored the use of SARS-CoV-2 genome sequences to understand the spread of the epidemic at the local [3], regional [4,5] or higher levels [6,7,8,9]. We present here an analysis of the sequences of 224 viral genomes collected in Madrid from February to May 2020 coincident with the first pandemic wave in our country. The sequences were uploaded to GISAID (https://www.gisaid.org/ (accessed on 3 February 2021)) and consequently some of them have been included in previous national [10] and global [11] analyses by other groups, but the analysis of the local dynamics of the epidemic in Madrid has not been done so far. Our aim is to understand which clades were initially introduced and successfully transmitted and spread in Madrid and the dynamic of replacement for those more prevalent clades at the end of the first pandemic wave. This approach might allow us to infer whether transmission was dominated by a particular lineage evolving locally during the lockdown period or by the clades previously introduced.

## 2. Materials and Methods

### 2.1. Sample Collection

We collected 224 samples from 211 patients for genome sequencing. Among them, 218 were obtained directly from oropharyngeal and/or nasopharyngeal swabs, 4 from Vero E6 cell culture supernatants (inoculated with nasopharyngeal samples) and 2 from respiratory secretions. The samples had been referred for diagnosis at three reference tertiary hospitals with more than 1100 beds in Madrid, Spain between 27 February and 27 May 2020. These hospitals cover different geographical areas of the city: Hospital Universitario La Paz (HLP) and Hospital Universitario Ramón y Cajal (HRYC) are located in the northern area of the city covering the rural and urban populations of the north of the Madrid region and distanced 1.5 Kms apart. Hospital Universitario 12 de Octubre (H12O) is 13 Kms away from the other two, located in the southern area of the city and covering part of the rural and urban populations of the south of the region. Detailed information regarding the samples and the patients is found in Table S1. Samples from hospitalized and non-hospitalized patients were included. For molecular diagnosis of SARS-CoV-2 infection, the samples were collected using flocked swabs and immersed in viral transport medium. The virus was inactivated by diluting 1:4 in 4M guanidinium thiocyanate containing 1 mg/mL of carrier RNA. Inactivated samples were stored at −80 °C until used. 

### 2.2. RNA Isolation and RT-PCR

Nucleic acid extraction was performed in each of the reference hospitals using automated systems. Detection of the SARS-CoV-2 RNA was done by RT-PCR using different commercial kits and real time PCR thermal cyclers. Positive and negative controls were included in each run, as well as internal controls to rule out the presence of PCR inhibitors in the samples. The threshold cycles (Ct) of the samples included in this study were retrieved from the respective hospital databases.

### 2.3. SARS-CoV-2 Genome Sequencing

cDNA was synthesized from the extracted RNA using the SuperScript IV VILO Master Mix (Thermo Fisher Scientific, Waltham, MA, USA) according to the manufacturer’s instructions. Briefly, 7 µL of sample were mixed with 2 µL of the 5X VILO solution and 1 µL of the 10X Superscript enzyme mix. The mixture was then incubated at 42 °C for 30 min and 85 °C for 5 min, and then stored at −80 °C until used. Targeted viral genome sequencing was carried out using oligonucleotide panels and two different technologies: Ion Torrent (Thermo Fisher Scientific, Waltham, MA, USA) and Oxford Nanopore (Oxford, UK). Libraries for whole-genome sequencing by Ion Torrent were prepared using the Ion AmpliSeq SARS-CoV-2 Research Panel (ThermoFisher Scientic, Carlsbad, CA, USA). This panel contains two primers pools targeting 237 amplicons (plus five human control sequences) with a length range of 125–275 bp covering > 99% of the SARS-CoV-2 genome. The libraries were constructed with the Ion AmpliSeq™ Library Kit using Ion Xpress™ Barcode Adapters (ThermoFisher Scientic, Carlsbad, CA, USA) and the intermediate purification steps were done using the Mag-Bind^®^ TotalPure NGS beads (Omega Bio-Tek, Norcross, GA, USA). After library preparation, total DNA was quantified using the Qubit dsDNA High Sensitivity Assay Kit (ThermoFisher Scientic, Carlsbad, CA, USA) and the Qubit 4 Fluorometer (ThermoFisher Scientic, California, USA). The libraries were normalized to 100 pM before pooling. The pools were quantified using the Ion Library TaqMan™ Quantitation Kit (ThermoFisher Scientic, Carlsbad, CA, USA) and the CFX Connect™ Real-Time System (BioRad, Marnes-la-Coquette, France), according to the manufacturers’ instructions. Then, 25 µL of the pools at 35 pM were loaded onto the Ion 530™ Chips using the Ion Chef™ Instrument (ThermoFisher Scientic, Carlsbad, CA, USA). The chips were then sequenced in an Ion GeneStudio S5 System (ThermoFisher Scientic, Carlsbad, CA, USA) using a built-in template for SARS-CoV-2 sequencing in the Ion Reporter™ Software.

For the Oxford Nanopore platform (Oxford, UK), cDNA, multiplex PCR reactions and sequencing were done according to the ARTIC nCoV-2019 sequencing protocol v2 [12]. The v1 CoV-2 primer panels (https://github.com/artic-network/artic-ncov2019/tree/master/primer_schemes/nCoV-2019/V1 (accessed on 3 February 2021)) were used for the two multiplex PCRs for SARS-CoV-2. The PCR products were end-repaired, adaptors and barcodes were ligated using the Ligation Sequencing Kit (SQK-LSK109; Oxford Nanopore Technologies, Oxford, UK). The libraries were then loaded on an R9.4 flow cell and sequenced on a MinION Mk1device (ONT). 

The sequences were deposited in GISAID (https://www.gisaid.org (accessed on 3 February 2021)) and ENA (https://www.ebi.ac.uk/ena/browser/home (accessed on 3 February 2021)). Accession numbers are shown in Table S2.

### 2.4. Bioinformatic Analyses

Base calling, trimming and quality control were done with the built-in pipeline in the Ion Reporter™ Software. The reads were then mapped against the reference genome Wuhan-Hu-1 (GenBank accession number: MN908947.3) using the IRMA assembler [13] plugin of the Ion Reporter™ Software (ThermoFisher Scientic, Carlsbad, CA, USA). Variant calling was done in parallel with the Variant Caller plugin of the Ion Reporter™ Software and with Snippy v.4.6.0 [14] (using default settings: 10 times minimum coverage and 90% minimum allele frequency). Annotations of variants were based on the reference genome. The Variant Caller plugin was used also to search for minor variants. To filter these, the reads were mapped to the reference strain using the Geneious Prime software (version 2020.0.4, Biomatters Ltd., New Zealand), and the variants were manually checked over the alignments. All the variants that had low coverage, were present in less than 15% of the reads, were found only near the ends of the reads (amplicons) or had low read qualities were discarded. Variants identified as problematic by De Maio et al. [15] were also discarded. 

The bioinformatic analysis for the Nanopore reads was based on the ARTIC pipelines. The open-source software RAMPART (version 1.0.5.2) [15] was used to assign and map reads in real-time. Raw files were basecalled with Guppy 1, demultiplexed and trimmed with Porechop and mapped against the reference strain Wuhan-Hu-1 (MN908947.3). Variants were called using Nanopolish.11.3 and accepted if they had a log-likelihood score of greater than 200. Low coverage regions were masked with N characters.

Lineages were assigned following the ’PANGO scheme [16] and using the Pangolin 2.0 web app (https://pangolin.cog-uk.io/ (accessed on 3 February 2021)), and the NextStrain year-letter scheme in the NextClade web page (https://clades.nextstrain.org/ (accessed on 3 February 2021)).

### 2.5. Phylogenetic Analysis

The 224 sequences obtained in this study, as well as the 58,888 SARS-CoV-2 genomes available on the GISAID database as of 2 July 2020, were aligned using MAFFT and then manually curated using MEGA X [17]. One sequence per patient was retained for further analyses. Genomes covering more than 90% of the entire genome were included in phylogenetic analysis (56,589 sequences). The best-fit nucleotide substitution model for the “Madrid dataset” (GTR + I) was identified according to the Akaike information criterion using jModelTest v2.1.10. Phylogenetic trees were reconstructed by ML with FastTree. Bootstrap values were estimated using the SH test (support > 95%). The bat betacoronavirus sequence RaTG13 (EPI_ISL_402131) was used as the outgroup. Monophyletic clades with support greater than 95% were identified as transmissions that may have occurred in Madrid.

Dated phylogeny was reconstructed using Bayesian inference through a Markov chain Monte Carlo (MCMC) framework implemented in BEAST v1.8.4 [18]. The days until the date of the most recent sequence were used as the sampling date. An uncorrelated relaxed clock model to estimate the time to a most recent common ancestor (TMRCA) was employed. Bayesian Skyline analysis was used to infer how the population size is expected to change over time. MCMC chains were run for 100 million steps with sampling every 10,000 steps from the previous distribution. Convergence was evaluated by calculating the effective sample sizes of the parameters using Tracer v1.7.1. All parameters had an effective sample size of more than 200, a number that is indicative of sufficient sampling. Trees were summarized as maximum clade credibility trees using TreeAnnotator v1.8.4 after discarding the first 10% as burn-in, and then visualized in FigTree v1.4.4.

## 3. Results

### 3.1. Description of SARS-CoV-2 Genomes Sampled among Patients with COVID-19 Diagnosis in Madrid during the First Pandemic Wave

In this multicentric study, 224 SARS-CoV-2 complete genomes corresponding to 211 patients were sequenced from clinical isolates obtained from three referral hospitals; 104 samples (from 101 patients) originated from Hospital 12 de October (H12O), 90 samples (from 80 patients) from Hospital La Paz (HLP), and 30 samples (from 30 patients) from Hospital Ramón y Cajal (HRYC). All the samples were collected during the first pandemic wave between 27 February and 27 May 2020. The mean age of the patients was 60.7 years (±21.1) and 121 of them (54.0%) were females. The average sequencing depth was 5878x (median 3873x). The age and genders of the patients, the origin of the samples, collection dates, assembly methods, and average depths are detailed in Table S1.

For the 202 samples sequenced with the Ion GeneStudio S5 System, intra-host diversity was analyzed looking for minority variants, defined as those mutations present in more than 15% and less than 50% of the reads. In 145/202 samples (70.4%), no minor variants were detected. Forty samples (20%) contained one minor variant, eight (4%) contained two variants and nine (4.4%) contained more than two variants. Three of the samples had minor variants that matched sequences detected in other samples and might be attributed to polyclonal infections or cross-contaminations. The remaining 54 were unique within the dataset, suggesting that they were intra-host mutations. In five patients, serial samples could be recovered from periods ranging 2 to 24 days. In most cases the different samples from the same patient had identical consensus sequences. The only exception was patient LP100. While the sample LP100-2 had a 12 bp deletion in genome positions 510-522, LP100-1 taken from the same patient three days later did not have the deletion. Examination of the sequencing reads showed that the two samples had mixed populations of the wild type and the deletion mutant sequences, the consensus sequence reflected the most frequent variant in each case.

**Figure 1:**
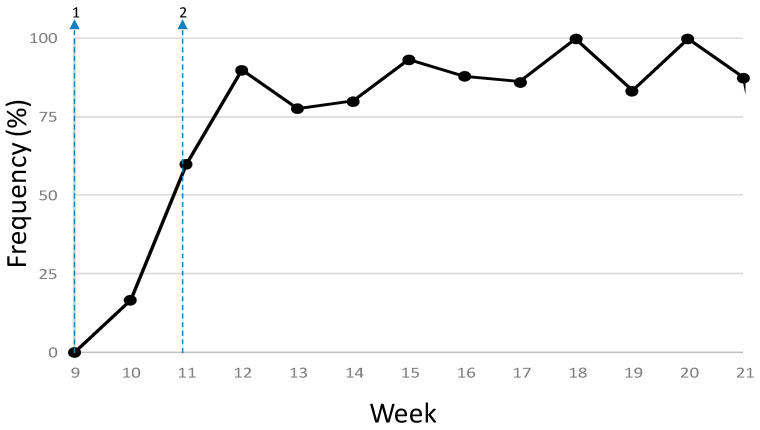
Frequency of the Spike protein D614G mutation among the sequenced samples. The arrows are 1, first case reported February 25th; 2, national lockdown, March 14th.

A total of 322 variants were identified among the 224 SARS-CoV-2 consensus sequences obtained in this study when compared to the reference strain (MN908947.3) [19], 250 (77.6%) of them appeared just once within the dataset. Each genome differed from the reference sequence in 2–13 nucleotide changes (1–9 amino acid changes), with an average of 7.1 SNPs per genome (between 3–4 amino acid changes). There were 17 mutations found in more than ten genomes, many of them are among the most frequently found globally [20,21], including a cluster of five mutations associated to clades 20A and 20B (lineage B and its sublineages): C241T (5’UTR, 78.1% of the samples), C3037T (nsp3 F924F, 79.5%), C14408T (nsp12 P4715L, 76.3%), A20268G (nsp15 L216L, 68.8%) and A23403G (Spike D614G, 79.5%). The D614G mutation in the Spike protein (A23403G) was detected in 16.7% of the samples from the first week (year-week 10), quickly increased in frequency up to 90% in the next two weeks and stayed over 80% until the end of the study period (Figure 1).

**Figure 2:**
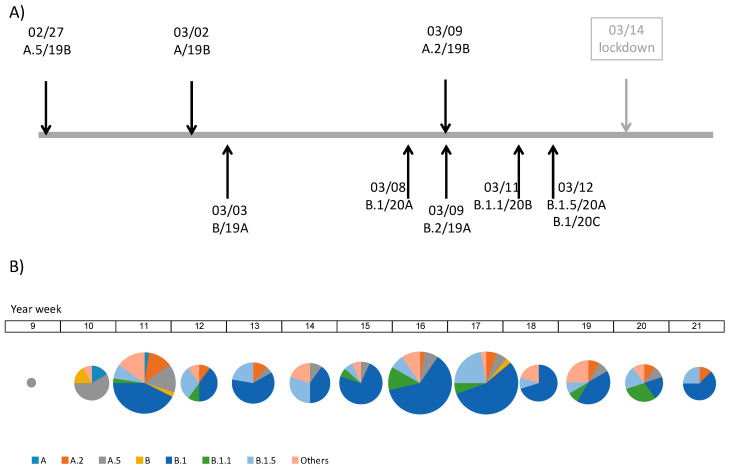
Timelines indicating (**A**) the dates (month/day) in which the major lineages/clades appear in our dataset and (**B**) the frequencies of the major lineages by weeks.

All the major clades described according to the different schemes in use were represented. Following the year-letter clade designation [19], 11 patients had viral genomes belonging to 19A (GISAID L/O/ and V clades), 41 to 19B (GISAID S clade), 146 to 20A (G clade), 13 to 20B (GR clade) and 3 to 20C (GH clade). One third of the 20A samples (52 out of 146) belonged to a variant characterized by the G29734C mutation in the 3’UTR region. Following the Rambaut scheme [16] (December 2020 update), the two major lineages, A and B, were found. The most frequent sublineages were A.2 and A.5 for lineage A, and B.1, B.1.1 and B.1.5 for lineage B. All the samples from the first and second week of the epidemic (year-weeks 9 and 10) belonged to clades 19A (Rambaut lineage B) and 19B (lineages A and A.5). In the third week (year-week 11) isolates belonging to clades 20A, 20B and 20C were identified for the first time (Figure 2A). In this same week clade 20A was already the most frequent and from there on remained the dominant clade for most of the study period.

H12O and HLP had very similar clade/lineage distributions, with 20A as the major clade and B.1 as the major lineage. In HRYC the major clade was 19B and the major lineage was A.5 (Table 1). This might be due to local differences but also to differences in the number of samples collected per hospital. In any case, the lineage composition of the dataset was relatively stable through time (Figure 2B).

### 3.2. Phylodynamics of SARS-CoV-2 in Madrid

A phylogenetic reconstruction was done including the sequences obtained in this study and 58,888 genomes downloaded from GISAID in the moment of this analysis (June 2020). The sequenced samples from Madrid were broadly distributed over the main branches of the global tree (Figure S1). 

**Figure 3:**
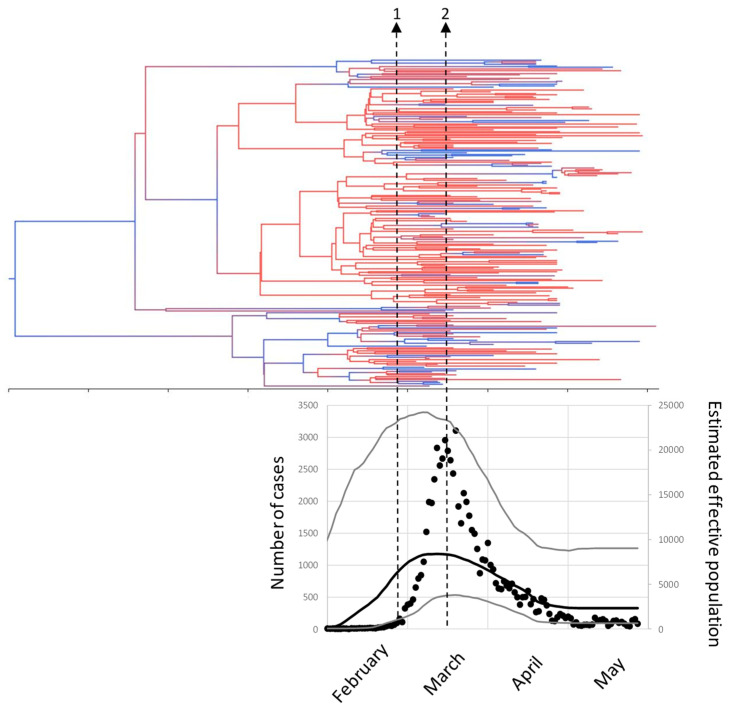
Phylodynamics of SARS-CoV-2 from Madrid. The tree shows the dated phylogeny. The tree is colored with a gradient from red (HPD = 0) to blue (HPD = 1). The plot below the tree is in the same time scale and shows the number of RT-PCR-confirmed cases in Madrid (black circles, left vertical axis) and the Bayesian skyline plot estimate of the effective population size (right vertical axis), the black line is the median estimate, and the grey lines indicate the 95% HPD interval. The arrows are (1) first case reported 25 February, (2) national lockdown, March 14th.

Dated phylogeny analysis located the most recent common ancestor of these sequences in October-November 2019, the major lineages diverged between November and January and most of our sequences diverged during February (Figure 3). Population dynamics were analyzed by the Bayesian skyline plot method (Figure 3) and showed that our sequences were obtained from a population growing fast since the beginning of February and reaching a maximum in the second week of March, matching the peak in the number of cases detected by RT-PCR. At the end of that week (March 14th) a national lockdown was declared and from there on the estimated effective population and the actual number of cases detected decreased gradually.

Potential transmission chains were searched using the sequences from the 211 patients. Fifteen monophyletic clusters, representing possible transmission events could be suggested (Table 2 and File S1). One of them was related to a nosocomial outbreak documented with epidemiological links between the subjects (labeled by an asterisk in Table 2). This cluster, from HLP, involved four patients from two adjacent rooms (LP77, LP78, LP81 and LP82) and one healthcare worker from the same ward (LP84). Sequencing identified another healthcare worker from an unrelated ward and no known link (LP51), and a patient from HRYC (R82) that had been admitted at HLP one week before, suggesting circulation of this clone within HLP and inter-hospital transmission. Other possible transmission chains were also detected (Table 2), but there were no known epidemiological links in these clusters. Eleven of them involved patients from a single hospital and four involved patients from two hospitals. Estimates of the diversification dates for these clusters agreed with the dates of the first reported cases and the median detection lag was 15 days (Table 2), indicating that local circulation of the virus did not start much before the first detected cases.

## 4. Discussion

We analyzed 224 SARS-CoV-2 genomes from respiratory samples collected in three Madrid hospitals during the first wave of the pandemic, from 27 February to 27 May 2020. By May 30th more than seventy thousand cases had been confirmed by RT-PCR in Madrid so our sample represents about 0.3% of all PCR confirmed cases in that period in our region. This study was designed to represent the major molecular features of the SARS-CoV-2 epidemic in Madrid, and to understand the viral dynamics of the first pandemic wave (introduction, spreading and shift). Moreover, Madrid is the Spanish city where the first unlinked cases were documented.

The analysis of intra-host variation showed that most samples were monomorphic. In addition, repeated samples from the same patients showed no sequence variations within the short periods of time observed (from three days up to four weeks). This low variability is in agreement with the fact that SARS-CoV-2, like all coronaviruses, accumulates mutations at a relatively slow pace [20], in contrast to other RNA viruses. As a consequence, during the first wave (February-May 2020) sequence diversity was still low, with an average of seven SNPs per genome in our dataset. This low sequence diversity complicated the analysis of phylogenetic relations among the different samples, although fifteen cases of transmission nodes with high statistical support could be identified. One of these was an epidemiologically supported nosocomial outbreak that identified a case of interhospital transmission, but for the other transmission nodes we did not have epidemiological information other than the dates and the collection sites. These samples had not been selected, and had been collected at hospital admission in a short span of time, suggesting that they were part of larger community transmission clusters.

The sequences from our sample were spread throughout the whole SARS-CoV-2 genome tree (Figure S1), spanning most major lineages defined by the different schemes [15,18]. All the major clades and lineages identified were detected for the first time before the lockdown (March 14th) and no new ones were detected after this date (Figure 2A). The first sequences detected (weeks 9 and 10) belonged to the most basal groups (clade 19A/lineage B and clade 19B/lineage A). These basal clades originated in China, though they already had a global distribution. The earliest sample included in the dataset (from February 27th) belongs to lineage A.5, proposed by Gómez-Carballa et al. to have originated in Madrid (haplogroup B.9 in [10]). In week 11, sequences belonging to lineages A.2 (19B), as well as B.1, B.1.1 and B.1.5 (clades 20A, 20B and 20C) were detected for the first time. Lineages A.2 and A.5 have been proposed to be of Spanish origin, though they were not abundant in our sample. The most frequent lineages were B.1, associated to the Italian outbreak [21], and B.1.5, a European lineage with probable Spanish origin (https://cov-lineages.org/lineages.html (accessed on 3 February 2021)). In week 11, appeared a B.1 subgroup that carries the mutation G29734C and has been proposed to arise in Madrid (haplogroup A2a5c in ref. [10]). This subgroup makes almost one third of the B.1 sequences.

The composition of the viral population detected in Madrid is different from that of the global Spanish population for the same period represented in the GISAID database and analyzed in references [8,10]. The frequency of clade 19B in our dataset was 18.3%, which is between the 1% reported in several European countries and the 40% reported in a global analysis of Spanish sequences [8]. On the other hand, the frequency of the D614G mutation was low during the first two weeks but quickly became dominant, being over 80% for most of the studied period, again in contrast with the publicly available Spanish data (around 50%) and similar to other European countries [8,10,22]. The rapid spread of this mutation might be related to higher transmission rates as suggested by some works [8,22], but the differences between Madrid and the publicly available Spanish data point rather to a strong founder effect generated by multiple introductions from European countries in the weeks before the lockdown. This is supported by the dated phylogeny and the effective population estimates, that show that an important diversification had already occurred before the first cases were detected in Madrid, and points to multiple entries from several sources. This would also make sense in the light of the role of Madrid as a national and international air transport hub, as it may have received the virus from different origins, and the viral composition might be a reflection of the different inputs.

A major limitation of this study is that the dataset represents only a small fraction of the number of cases detected by RT-PCR in the period studied. Moreover, PCR testing was mostly restricted to symptomatic patients at that time, so the actual fraction would be still lower, as would be according to a national seroepidemiological study [23]. Another problem might be that not all the sequences had been randomly selected, and they do not cover the whole geographic range of the Madrid region. In spite of these limitations, the data provide an image of the first epidemic wave in Madrid. The pattern of multiple and almost simultaneous virus entries from different origins suggested by the data is similar to those that have been described in several places and at different scales during this pandemic [3,4,5,6,9]. The lineage composition departs from the publicly available Spanish data and reflects the role of Madrid as a national and international travel and transportation hub, and the strong connections with European countries.

## Figures and Tables

**Table 1:** Distribution of the 224 genomes sequenced in this study according to hospital and clade (Nexstrain year-letter clade nomenclature). Numbers in parenthesis are percentages per columns. H12O: Hospital Universitario 12 de Octubre; HLP: Hospital Universitario La Paz; HRYC: Hospital Universitario Ramón y Cajal.

Clade	H12O	HLP	HRyC	Total
19A	6 (5.8)	5 (5.6)	0 (0)	11 (4.9)
19B	14 (13.5)	13 (14.4)	14 (46.7)	41 (18.3)
20A	76 (73.1)	68 (75.6)	11 (36.7)	155 (69.2)
20B	5 (4.8)	4 (4.4)	5 (16.7)	14 (6.3)
20C	3 (2.9)	0 (0)	0 (0)	3 (1.3)
Lineage				
A.2	7 (6.7)	5 (5.6)	2 (6.7)	14 (6.3)
A.5	7 (6.7)	7 (7.8)	10 (33.3)	24 (10.7)
B.1	60 (57.7)	49 (54.4)	3 (10)	112 (50.0)
B.1.1	5 (4.8)	4 (4.4)	5 (16.7)	14 (6.3)
B.1.5	15 (14.4)	11 (12.2)	5 (16.7)	31 (13.8)
Others	10 (9.6)	14 (15.6)	5 (16.7)	29 (12.9)

**Table 2:** Transmission clusters with bootstrap support >95% identified by the dated phylogeny. The table shows for each cluster the estimated diversification date (date of the most recent common ancestor) and its 95% HPD interval, the date of the first sequenced sample belonging to the cluster and the number of days between the diversification estimate and the first sequenced sample (detection lag). The asterisk indicates a nosocomial transmission cluster. See also File S1.

Transmission Node	Diversification Date	95% HPD Interval		First Sequenced Sample	Detection Lag (Days)
H12_57/LP55/LP88/H12_2208/LP57/LP68	25/02/2020	29/01/2020	20/03/2020	27/03/2020	32
LP19/LP22	29/02/2020	17/01/2020	07/03/2020	07/03/2020	7
R64/H12_45/H12_59	02/03/2020	10/02/2020	17/03/2020	17/03/2020	15
H12_28/H12_29	05/03/2020	19/02/2020	12/03/2020	12/03/2020	7
H12_54/H12_70/ H12_71/H12_66	05/03/2020	09/03/2020	11/04/2020	01/04/2020	26
H12_43/H12_56/ H12_85	10/03/2020	11/02/2020	05/04/2020	06/04/2020	26
R62/LP105	14/03/2020	09/02/2020	15/04/2020	21/04/2020	37
LP90/LP100	16/03/2020	12/02/2020	15/04/2020	26/04/2020	40
H12_47/H12_88/ H12_55	20/03/2020	19/02/2020	09/04/2020	09/04/2020	19
H12_42/H12_81	26/03/2020	03/03/2020	05/04/2020	06/04/2020	10
H12_63/H12_64/ H12_62	29/03/2020	09/03/2020	12/04/2020	12/04/2020	13
H12_82/H12_83	30/03/2020	05/03/2020	16/04/2020	16/04/2020	16
H12_72/H12_73	03/04/2020	12/04/2020	21/05/2020	13/04/2020	10
* R82/LP77/LP78/LP82/LP84/LP51	03/04/2020	09/03/2020	20/04/2020	05/04/2020	2
R56/R84	05/04/2020	13/03/2020	20/04/2020	20/04/2020	15

## Data Availability

The sequences were deposited in GISAID (https://www.gisaid.org, accessed on 17 February 2021) and ENA (https://www.ebi.ac.uk/ena/browser/home, accessed on 17 February 2021). Accession numbers are shown in Table S2.

## Supplementary Materials

The following are available online at https://www.mdpi.com/2076-2607/9/2/454/s1, Figure S1: SARS-CoV-2 phylogenetic reconstruction using Maximum-likelihood. Table S1: Sample and patient data. Table S2: Sample lineages, clades, mutations, and accession numbers. File S1. Image of the dated consensus tree indicating the transmission nodes in blue.
