# Supplementary material for: Genomic Epidemiology of SARS-CoV-2 in Madrid, Spain, during the First Wave of the Pandemic: Fast Spread and Early Dominance by D614G Variants
Source: Microorganisms. 2021 Feb 22;9(2):454. doi: 10.3390/microorganisms9020454 (PMC7926973; doi:10.3390/microorganisms9020454)
Supplement: Supplementary file 1 [file microorganisms-09-00454-s001.zip › Suppl Final/Suppl final.docx]

**Figure S1.** SARS-CoV-2 phylogenetic reconstruction using Maximum-likelihood (GTR + I, SH≥95). BetaCoV/Bat/Yunnan/RaTG13 was included as an outgroup. The circles indicate the sequences of our study: white circles are sequences obtained before the national lockdown and black circles are sequences obtained after the national lockdown.

**File S1.** Image of the dated consensus tree indicating the transmission nodes in blue.
