# Supplementary figures and images for: Genomic Epidemiology of SARS-CoV-2 in Madrid, Spain, during the First Wave of the Pandemic: Fast Spread and Early Dominance by D614G Variants
Source: Microorganisms. 2021 Feb 22;9(2):454. doi: 10.3390/microorganisms9020454 (PMC7926973; doi:10.3390/microorganisms9020454)

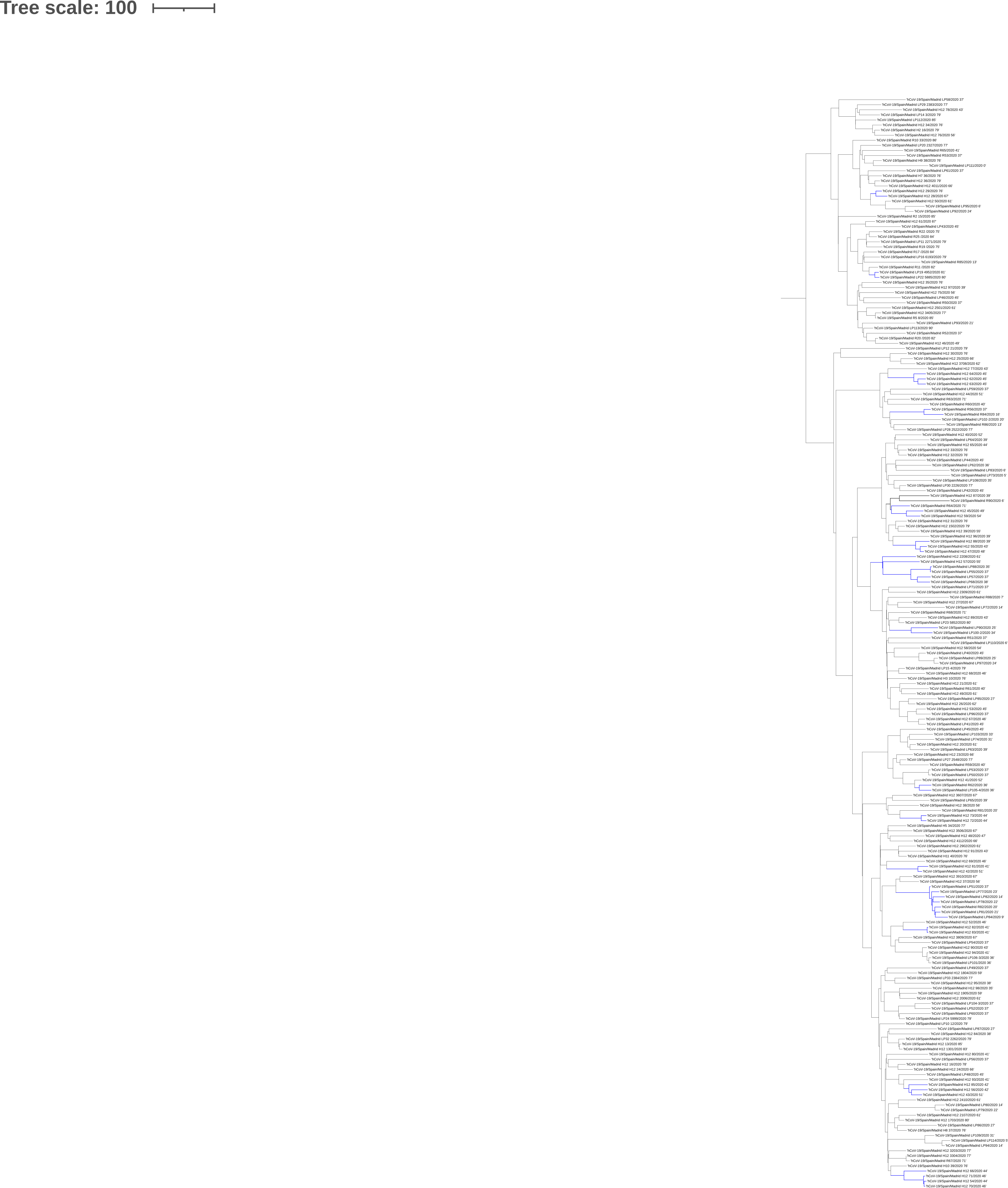

Supplement: Supplementary file 1 [file microorganisms-09-00454-s001.zip › Suppl Final/Supp. File 1.png]
